# Value of ultrasound-guided irrigation and drainage of refractory pyocysts in ADPKD

**DOI:** 10.1186/1757-1626-2-66

**Published:** 2009-01-20

**Authors:** Daryoush Saedi, Peyman Varedi, Payam Varedi, Simin Mahmoodi, Hossein Nejad Gashti, Mohsen Darabi

**Affiliations:** 1 Department of Radiology, Hasheminejad Kidney Center, School of Medicine, Iran University of Medical Sciences, Valiasr Ave, Vanak Sq, 19697, Tehran, Iran; 2 Department of Nephrology, Hasheminejad Kidney Center, School of Medicine, Iran University of Medical Sciences, Tehran, Iran; 3 Department of Dentistry, Faculty of Dentistry, Tehran Islamic Azad University, Tehran, Iran

## Abstract

**Background:**

Cyst infections is not common in the patients with autosomal dominant polycystic kidney disease (ADPKD) however it may pose major problems to the clinicians because the diagnosis is hampered by lack of reliable imaging techniques for identification of the infected cysts and treatment may be difficult due to poor penetration of antibiotics into the cysts.

**Case presentation:**

We present a case of ADPKD and intractable pyocysts that did not respond to standard antibiotic therapy but successfully treated by using ultrasound-guided cyst puncture, and repeated irrigation and drainage.

**Conclusion:**

Where the experienced interventional radiologists are available, this method can rescue these patients from nephrectomy.

## Background

Urinary tract infection (UTI) occurs frequently in the patients with autosomal dominant polycystic kidney disease (ADPKD). Among the various forms of UTI, infections involving the cysts are usually diagnostic dilemmas and often refractory to therapy possibly because of poor penetration of antibiotics into the cysts [1]. Herein, we present a case of ADPKD with refractory pyocysts that was successfully treated by using an interventional method. The feasibility and usefulness of interventional radiology in the management of refractory pyocysts in the patients with ADPKD will be discussed.

## Case presentation

**Figure 1:**
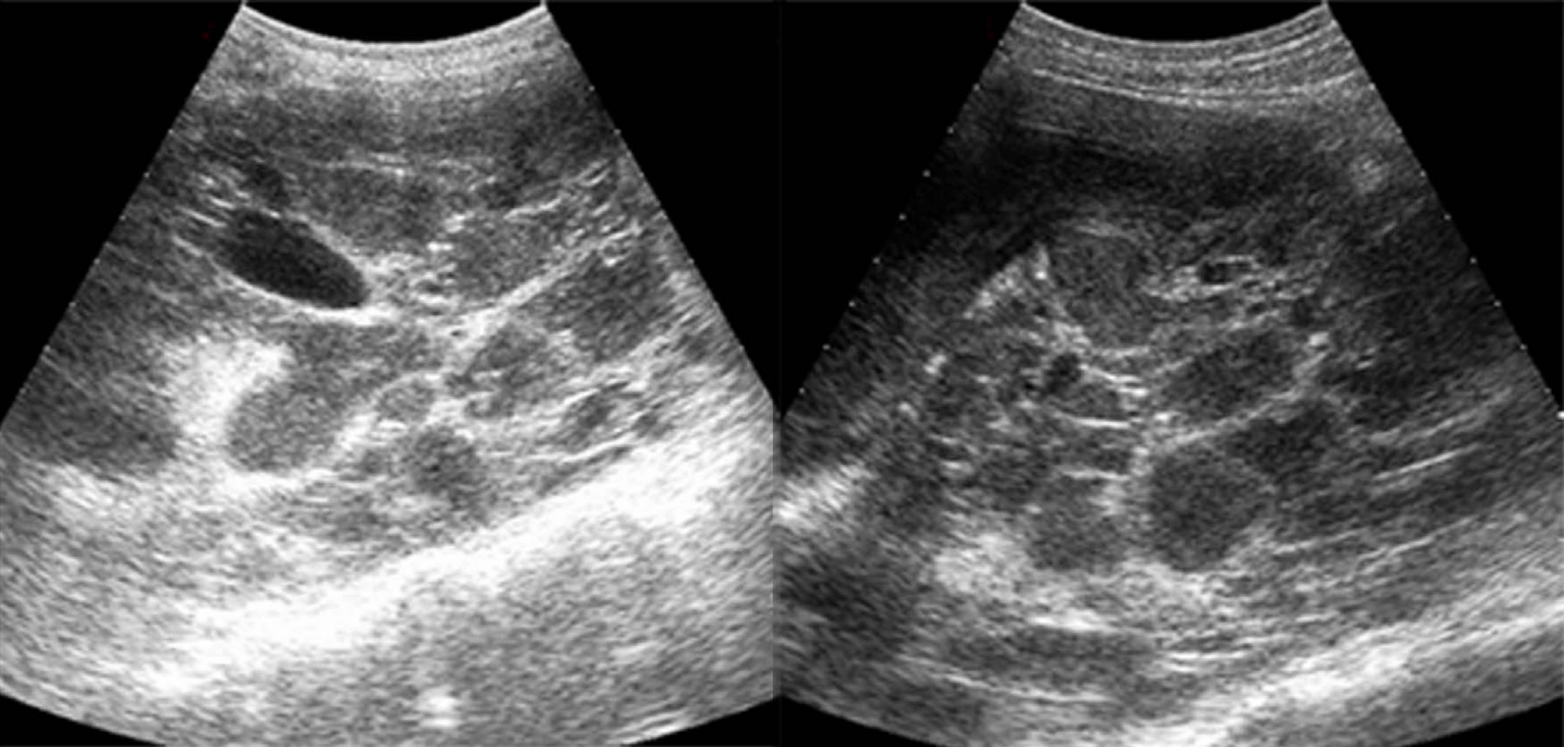
**Sagittal gray-scale ultrasound scan of both kidneys obtained before intervention shows numerous cystic space-occupying lesions with marked internal echogenic debris and thick septa representing the numerous infected cysts**.

**Figure 2:**
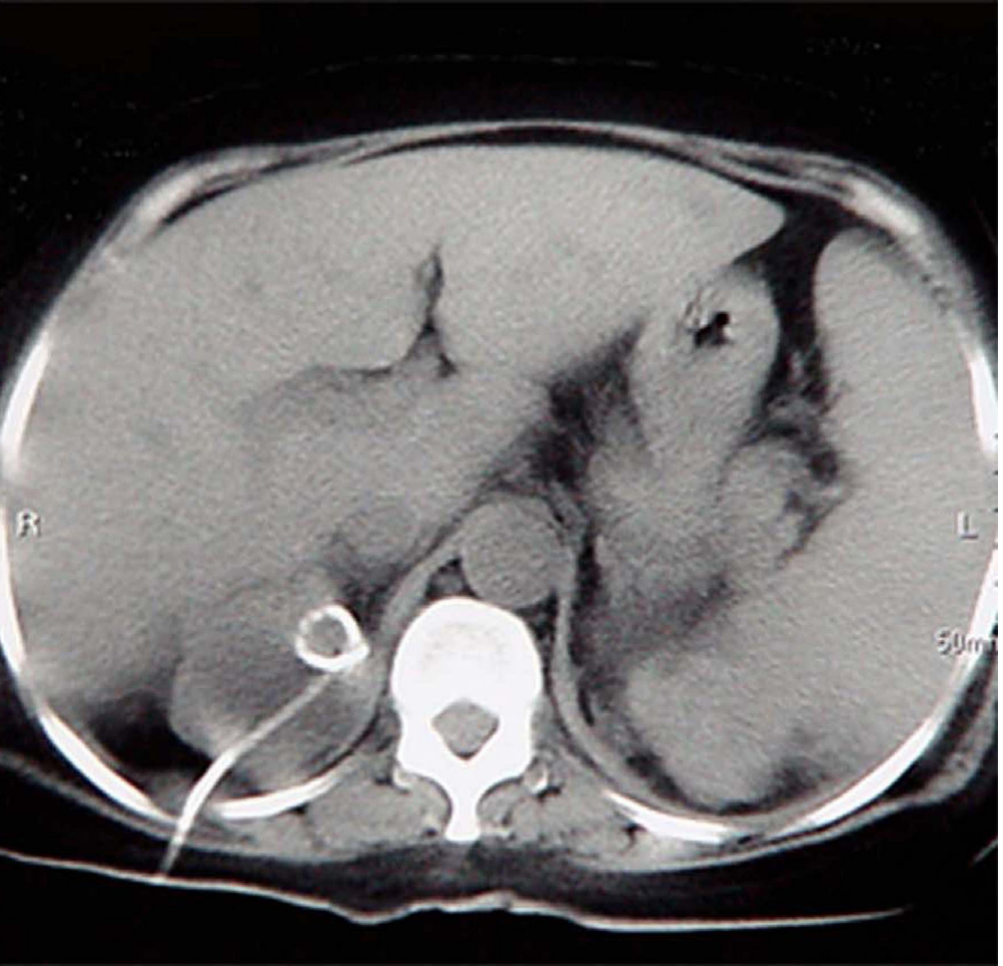
**Axial CT scan of both kidneys without contrast demonstrates the nephrostomy catheter which was in the right kidney for further irrigation and drainage of the remaining pyocysts**.

**Figure 3:**
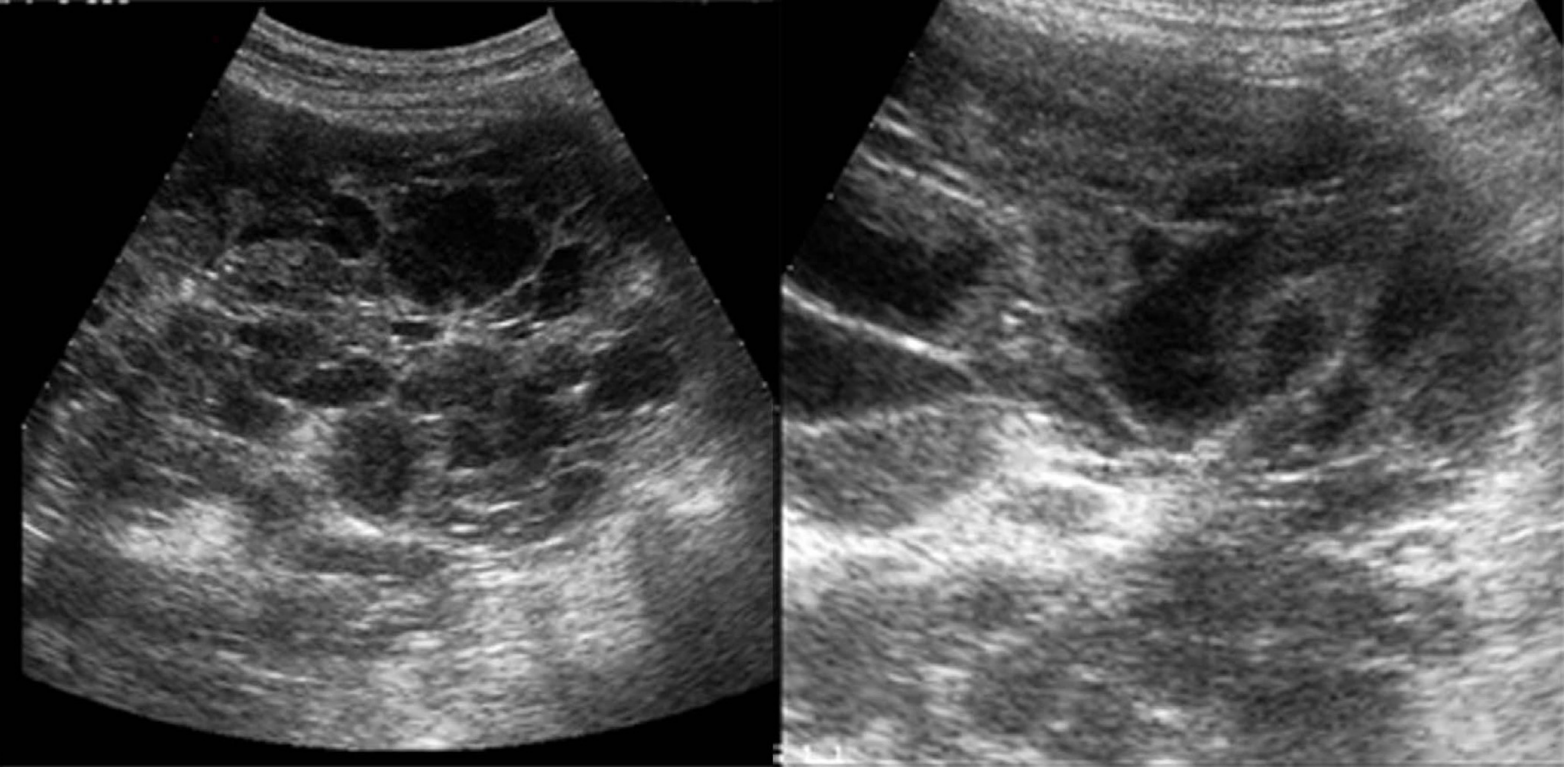
**Sagittal gray-scale ultrasound scan of right kidney obtained after repeated irrigation and drainage demonstrates decreasing amount of internal echoes and septa in the cysts**.

A 55-year-old Iranian woman (height: 168 cm weight: 78 Kg), a known case of ADPKD, was admitted to our hospital with 3-week history of malaise, severe bilateral flank pain, dysuria, frequency, chills, and fever. She had also pertinent past medical history of diabetes mellitus and hypertension for 8 years and was receiving atenolol 50 mg and glibenclamide 25 mg daily. She had neither history of cigarette smoking nor alcohol consumption. Her family history was unremarkable. She had three normal full term pregnancy without any complications. On physical examination, she was febrile and ill. Mild pretibial edema and tenderness over the costovertebral angles were also noted. Laboratory studies showed anemia, elevated level of blood urea nitrogen (BUN), serum creatinine, and serum glucose. Urinalysis showed pyuria, and urine culture yielded growth of Escherichia coli and Pseudomonas aeroginosa (more than 10^5 ^colonies per high power field). Hence, antibiotic therapy was begun with the administration of ciprofloxacin intravenously. Abdominal Ultrasonography (US) demonstrated both enlarged kidneys measuring 18 cm on the right side and 23.6 cm on the left contained multiple cysts. The largest cysts in the right and left kidney were 8 cm and 10 cm respectively, with most of the remaining cysts measuring 4 to 5 cm. A renal calculus was also seen in the left pelvicalyceal system. No hepatic cyst was noted. Due to the lack of clinical improvement, intravenous piperacillin/tazobactam was added to ciprofloxacin on the 5th day of her admission. Further US investigation on the 12th day, showed numerous cystic space-occupying lesions with low level internal echoes, septations, and increased wall thickness of the cysts that representing infected cysts (Fig. 1). So, intravenous trimethoprim-sulfamethoxazole (TMP-SMX) was added to the former antibiotic therapy. This 2-week intensive antibiotic therapy had no beneficial effect. After informing the patient and her family about her condition and obtaining a signed consent, the antibiotics were discontinued and then we decided to perform puncture and irrigation of the cysts for confirming the diagnosis of pyocyst, determining the causative organisms, and treating the refractory infected cysts. After sterile preparation, US-guided percutaneous puncture was carried out by using a 20-gauge Chiba needle (TSK, Japan) in the supine position and 40 ml of purulent fluid was evacuated and sent for microbiological and serological studies. The culture was positive to E. coli and Pseudomonas aeroginosa. On the second stage, the largest cysts in the left side were punctured by an identical method. Cystic fluid was aspirated as completely as possible. To achieve this, the needle tip was monitored with US so that it remained in the center of the cyst during aspiration. Immediately after that, an 8 Fr. Catheter was inserted into the cystic cavity with US guidance and irrigation and drainage with 20 ml of 0.9% sodium chloride solution, and 20 ml of 10% povidone iodine was carried out. Forty-eight hours later, the same procedure was repeated for the largest cyst (8 cm in diameter) of the right kidney (Fig. 2); however, the patient showed no improvement. Despite the repeated irrigation and drainage via the inserted catheters, no response to the procedures was seen. Therefore we decided to schedule her for bilateral nephrectomy on the 23rd day, but she didn't consent to the operation. We continued irrigation of catheters with 10 ml of 0.9% saline solution every 8 hours for removing the infected debris and draining the purulent discharge. Ultimately, a catheter was inserted into another large cyst of the left kidney for irrigation and drainage. After 3 days, fever disappeared and clinical state and laboratory tests showed improvement. Repeated US showed decreasing amount of internal echoes and thick septa of the drained cysts (Fig. 3). Two catheter in the left and one in the right side were removed once the amount of drainage became less than 10 ml and sterile. Then, antibiotic therapy was appropriately changed to oral TMP-SMX. After 2 months of hospitalization, she completely recovered and was discharged to home. One year after discharge, she remained asymptomatic.

## Discussion

The prevalence of UTI in the ADPKD is abnormally high. Infections involving the cysts are often refractory to the antibiotic therapy because of limited entry of commonly used antibiotic into the cysts. Treatment of infected cysts in ADPKD should begin with lipophilic compounds such as ciprofloxacin and new quinolones, TMP-SMX, because of their low toxicity and high volume of distribution [1-4]. In the lack of response to the antibiotic therapy, cyst drainage may be a suitable alternative method. The successful use of irrigation drains for the treatment of the cyst infection in ADPKD subjects has been reported scarcely in the literature [1-3]. It should be considered that the identification of infected cysts is essential for imaging-guided diagnostic or therapeutic percutaneous puncture. US may be a feasible method for the evaluation of complicated cysts in ADPKD however it often fails to reliably differentiate benign hemorrhagic cysts from the other complications [4]. Chapman et al. [1] suggested that percutaneous drainage under fluoroscopic localization should be the treatment of choice for the accessible infected cysts greater than 5 cm in diameter. The interesting point in the management of this case was the effectiveness of repeating this procedure despite the lack of response in the initial stages. We stress that even in severe cases with persistent and refractory pyocysts which shows no response to the initial stages of irrigation and drainage, repeated irrigation and drainage in a logical sequence will be an appropriate method for rescuing the patient from nephrectomy. This observation is in contrast with the former studies which have recommended nephrectomy in the severe cases, or in those with persistent recurrent infections. One problem still to be resolved is the role of intracystic irrigation by using the antibiotics and/or povidone iodine. To our knowledge, no evidence based data about the efficacy of this method in the treatment of intractable pyocysts in ADPKD has been reported in the English literature so far. Povidone iodine as a sclerosing agent was successful in the ablation of giant renal cysts [5], but no data about the beneficial effects of this agent in the treatment of pyocyst has been mentioned. Clearly, determining the role of irrigation of the infected cysts with the ideal antibiotics such as quinolones and/or povidone iodine requires replication in other studies. Hence, the success of this treatment may be related to the irrigation and drainage with saline solution solely. On the other hand it should be noted that iodine irrigation can increase the blood iodine levels significantly. We used the povidone iodine as an irrigating agent only in the first stage and we didn't measure the level of plasma iodine in the current patient however no aggravation of renal function and another complication was occurred. We emphasize that plasma iodine levels should be monitored in the patients with renal insufficiency; because iodine toxicity can lead to increased morbidity and mortality of these patients. In conclusion, this method is an effective and feasible method for treatment of the patients with ADPKD and intractable pyocysts and therefore, where experienced interventional radiologists are available, this method can rescue these patients from nephrectomy.

## Competing interests

The authors declare that they have no competing interests.

## Authors' contributions

DS has performed the procedure and contributed in the preparation of the clinical data and laboratory investigation. HNG has participated in the treatment of the case and informed the family of the patient regarding the efficacy of the procedure. He also participated in the procedure with DS and collected the laboratory data and clinical findings. PV and MD have contributed in the writing of the case report and the discussion. PV and SM have contributed in the reviewing of the literature about the efficacy of the cyst drainage in the treatment of the refractory pyocysts of the APKD and other aspects of the treatment of UTI in the patients with ADPKD.

## Consent

Written informed consent was obtained from the patient for publication of this case report and accompanying images. A copy of the written consent is available for review by the Editor-in-Chief of this journal.
